# Recent advances in synthetic and medicinal chemistry of phosphotyrosine and phosphonate-based phosphotyrosine analogues

**DOI:** 10.1039/d0md00272k

**Published:** 2020-10-15

**Authors:** Nikolai Makukhin, Alessio Ciulli

**Affiliations:** Division of Biological Chemistry and Drug Discovery, School of Life Sciences, University of Dundee Dow Street DD1 5EH Dundee UK a.ciulli@dundee.ac.uk

## Abstract

Phosphotyrosine-containing compounds attract significant attention due to their potential to modulate signalling pathways by binding to phospho-writers, erasers and readers such as SH2 and PTB domain containing proteins. Phosphotyrosine derivatives provide useful chemical tools to study protein phosphorylation/dephosphorylation, and as such represent attractive starting points for the development of binding ligands and chemical probes to study biology, and for inhibitor and degrader drug design. To overcome enzymatic lability of the phosphate group, physiologically stable phosphonate-based phosphotyrosine analogues find utility in a wide range of applications. This review covers advances over the last decade in the design of phosphotyrosine and its phosphonate-based derivatives, highlights the improved and expanded synthetic toolbox, and illustrates applications in medicinal chemistry.

## Abstract 2

This review summarizes advances over the last decade in the design of phosphotyrosine and its phosphonate-based derivatives, focusing on their synthesis and medicinal chemistry applications.

## Graphical abstract

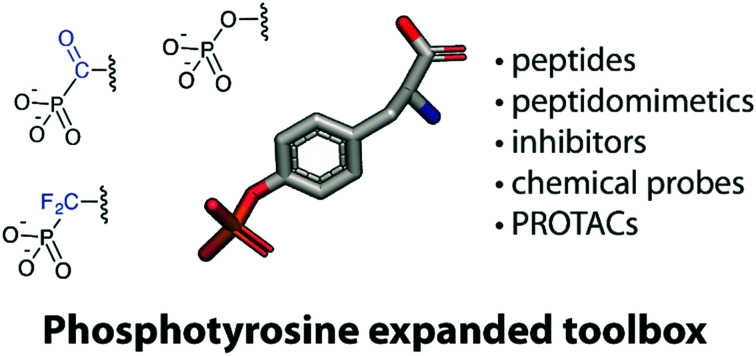

## Introduction

Reversible protein phosphorylation is one of the most important cellular events as it plays a crucial role in signal transduction, activation or deactivation of genes transcription, and on/off control of enzyme activity.^1,2^ Although protein tyrosine phosphorylation occurs at low level compared to serine or threonine phosphorylation, protein tyrosine phosphorylation is vital for cell growth regulation, mitogenesis, metabolism and apoptosis.^3^ The main steps of this pathway involve the phosphorylation of tyrosine by protein tyrosine kinases (PTKs), recognition of pY residues by proteins containing pY-binding modules such as Src homology 2 (SH2) or phosphotyrosine binding (PTB) domains, and removal of a phosphotyrosine phosphate group by protein tyrosine phosphatases (PTPs). Misfunctions in each of these steps have been linked to numerous diseases such as inflammation, diabetes, cancer and metabolic and autoimmune diseases.^4–8^

The significance of protein tyrosine phosphorylation motivates the development of pY containing peptides and small-molecule derivatives that can mediate protein–protein interactions or serve as substrate/product mimetics, thereby providing useful chemical tools to study the roles of individual proteins involved in signalling pathways or pathways contributing to diseases.^9–13^ Such compounds can also provide useful tools for biophysical protein characterization,^14^ for monitoring enzyme activity,^15,16^ and as antigens for raising antibodies.^17,18^ Moreover, synthetic pY-containing peptides have been used to obtain co-crystal structures of protein–peptide complexes,^11,14,19,20^ revealing structural insights into substrate recognition and specificity of the enzymes, and guiding structure-based drug design. Additionally, the immobilisation of pY on agarose gel has allowed the purification of human immunoglobulin G (IgG)^21^ and pre-miRNA (MicroRNA)-29 (ref. 22) from human plasma *via* mimicking the natural interactions. Due to hydrolysis of the phosphate group by PTPs, pY-containing macromolecules have found interesting applications in phosphatase-instructed self-assembly to form higher-order nanostructures,^23,24^ which can be used in the design of probes for biomedical imaging applications^25^ and enhanced photoacoustic imaging of tumours.^26^

Phosphotyrosine residues and analogues are challenging functional groups for drug design because of their negative charge at physiological pH and the enzymatic lability of the phosphate group. The first limitation can be solved by applying a prodrug approach.^27^ The improvement of the metabolic stability in the presence of PTPs has been a challenging task for medicinal chemistry and a whole discipline has arisen devoted to the design of pY mimetics.^28,29^ After significant research phosphonodifluoromethyl phenylalanine (F_2_pmp) was found to be one of the most promising physiologically stable pY analogues. Recently, the F_2_pmp scaffold was successfully employed in the discovery of cell-permeable PTP inhibitors^30–32^ and for affinity purification of SH2 proteins from cell lysates.^33^

Targeting of phosphotyrosine-binding domains by small molecules has attracted particular attention over the last decades, and is now seeing a renaissance as a promising approach in drug discovery.^6,9,34^ The syntheses of pY containing molecules and pY mimetics are well established and have been previously reviewed.^35,36^ Here we summarise approaches developed over the last ten years and highlight methods not previously discussed.

## Structural basis of phosphotyrosine recognition

Phosphotyrosine is ‘read’ by cellular proteins binding tightly to the pY side chain phosphate group with high specificity. However, the structural details of the molecular recognition differ depending on the domain being used.

**Fig. 1:**
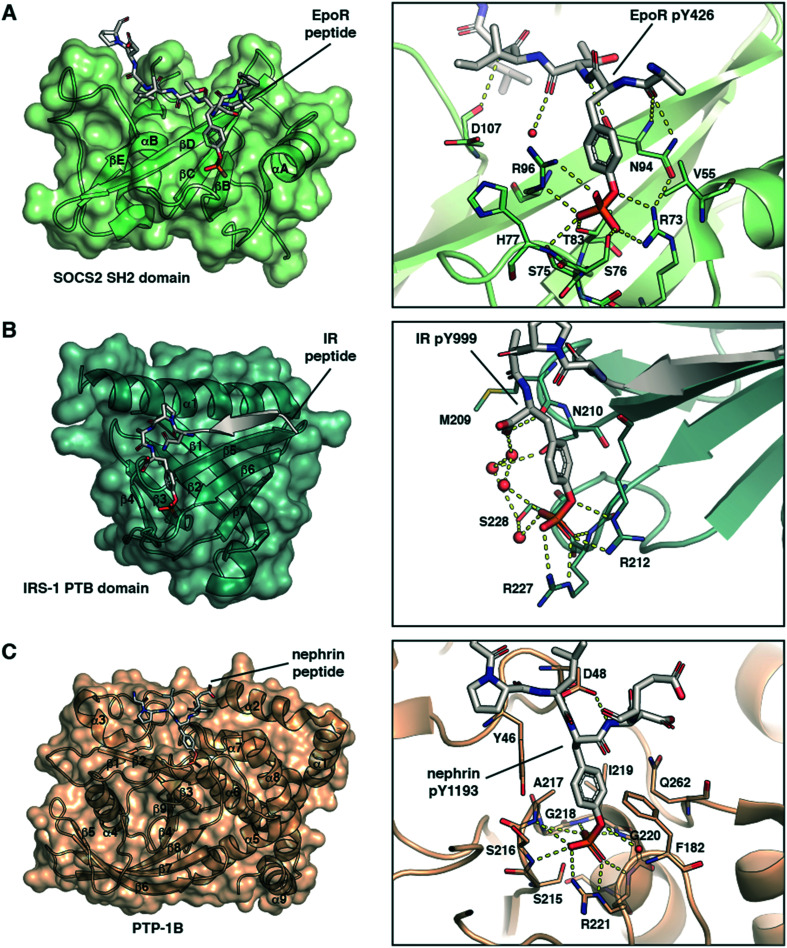
Structural basis of molecular recognition specificity of pY-reader domain interactions. Left: Architecture of the reader domain, with secondary structure elements labelled. Right: Zoom-in on the binding mode and hydrogen bond interactions between the pY-substrate peptide and the domain pY-binding pocket. A) SH2 domain recognition. Crystal structure of SOCS2 in complex with phosphorylated EpoR peptide (ASFEpYTILDPS-amide, PDB code: 6I4X).^14^ Only the SH2 domain of SOCS2 is shown. B) PTB domain recognition. Crystal structure of the IRS-1 PTB domain bound to the juxtamembrane region of the insulin receptor (LYASSNPApY, PDB code: 5U1M).^42^ C) PTP domain recognition. Crystal structure of PTP-1B catalytically inactive mutant C215S in complex with nephrin peptide (AWGPLpYDEVQM, PDB code: 4ZRT).^43^

The SH2 domain is a structurally conserved reader domain whose general domain architecture and pY recognition mode are well understood following decades of studies since its first discovery.^37–39^ The SH2 domain comprises multiple β-sheets flanked by two α-helices (Fig. 1A, left), and it has two distinct regions. The first region, highly conserved within the family, contains the pY-binding pocket. This pocket is located at the N-terminal region between the first α-helix and the central β-strand where the pY phosphoryl group interacts with two positively charged Arg residues and forms additional hydrogen bonds with Ser or Thr residues (Fig. 1A, right). Of note, this is not the case with all SH2 domains. For example, the SH2 domains of STAT proteins can engage the phosphate group with a single Arg residue.^40^ The second part of the domain, defined between the central β-strand and the C-terminal α-helix, provides specificity for substrate binding *via* interactions with residues C-terminal to pY in the peptide binding epitope. The recognition mode of SH2 domains for pY partner proteins thus involve anchoring of pY to accommodate the flanking regions of the peptide on the domain surface. Our group recently solved co-crystal structures of the SH2-containing E3 ligase substrate receptor subunit suppressor of cytokine signalling 2 (SOCS2) in complex with pY-modified peptides from substrates growth hormone receptor (GHR) or erythropoietin receptor (EpoR) (Fig. 1A).^14^ The structure illustrates the canonical SH2-pY recognition mode, and identifies the C-terminus of the peptide as fitting snugly through the C-terminal hydrophobic cavity of the domain, forming hydrogen bonds from its backbone amide bonds along with hydrophobic interactions.

PTB domains represent another modular unit that recognize pY-containing proteins. The overall structure of the domain and the substrate recognition features are different from the SH2 domain. PTB domains have a conserved fold consisting of two central orthogonal β-sheets (made of seven β-strands overall), capped by a carboxy-terminal α-helix (Fig. 1B, left).^39^ Unlike SH2 domains, PTB domains bind to partner proteins by recruiting amino acids N-terminally to the pY residue. They recognise an NPXpY sequence motif, and the pY binding pocket is typically solvent-exposed. Of note, a phosphorylated tyrosine residue is not required for high-affinity binding to all PTB domains and in some cases even inhibits it.^37^ However, mutations at that position, for example Y-to-E or Y-to-F, typically abolish the protein–protein interaction and inhibit biological activity.^41^ The crystal structure of the PTB domain of the insulin receptor substrate 1 (IRS-1) bound to a phosphorylated peptide from the juxtamembrane region of the insulin receptor (IR) illustrate these recognition features (Fig. 1B).^42^ pY forms salt-bridge hydrogen bonds with two Arg residues and with a network of water molecules (Fig. 1B, right). The N-terminus stretch of the peptide forms a β-strand that fits snugly into a groove between the α-helix and the β5-strand of the domain (Fig. 1B, left).

Protein tyrosine phosphatases (PTPs) are a third class of proteins that recognize pY-containing substrates in order to catalyse their dephosphorylation. This catalytic process relies on the presence of a cysteine residue located in the PTP phosphate binding site, defined by the sequence HCX_5_R.^37^ This motif is strictly conserved among the PTP family members. The stabilization of the pY phosphate group is achieved by an Arg residue, that together with the backbone amide nitrogens of the CX_5_R motif forms a ‘crown-like’ coordination mode (Fig. 1C).^44^ Selectivity for pY over phosphothreonine (pT) and phosphoserine (pS) is established by the depth of the active site and by the presence of specific aromatic residues at the mouth of the pocket. While the catalytic mechanism of PTPs is well established, substrate specificity remains elusive for numerous phosphatases. To this end, pY-containing peptides provide useful chemical tools to study the protein–protein interaction and gain insights into substrate specificity. The crystal structure of PTP-1B protein bound to a phosphorylated peptide from substrate nephrin illustrate these recognition features (Fig. 1C, left).^43^ The nephrin peptide binds to PTP-1B in a canonical fashion with its pY residue anchored tightly by hydrogen bond interactions, and it phenyl group “sandwiched” *via* π-stacking interactions. The nephrin peptide makes additional hydrogen bond interactions beyond pY along the backbone (Fig. 1C, right).

The structures presented in Fig. 1 are representative examples highlighting the recognition of the pY group by its reader domains. Regions flanking the pY residue are also important for the recognition of pY-containing substrates by these reader proteins, and as such they contribute to substrate specificity as reviewed previously.^37,39^ Notwithstanding the complexity of the molecular recognition beyond pY, the structures illustrate the significance of the double-negative charge and the ester oxygen of the phosphate group for high binding affinity of pY and pY-mimetic ligands. These observations were important to the development of phosphonate-based phosphotyrosine mimetics and led to a discovery of F_2_pmp.^28,29^ Importantly, the –CF_2_– group has larger size than the ester –O– group, and the sum of the bond lengths within the fragment P–CF_2_–C (P–CF_2_ bond plus CF_2_–C bond) is greater than for the P–O–C fragment (P–O bond plus O–C bond). These structural features can affect the ligand binding mode and lead to a loss in binding energy. However, during the ligand design this loss in the binding affinity can be compensated and so recouped by optimizing the rest of the molecule.

## Advances in the synthesis of phosphotyrosine-containing compounds

**Fig. 2:**
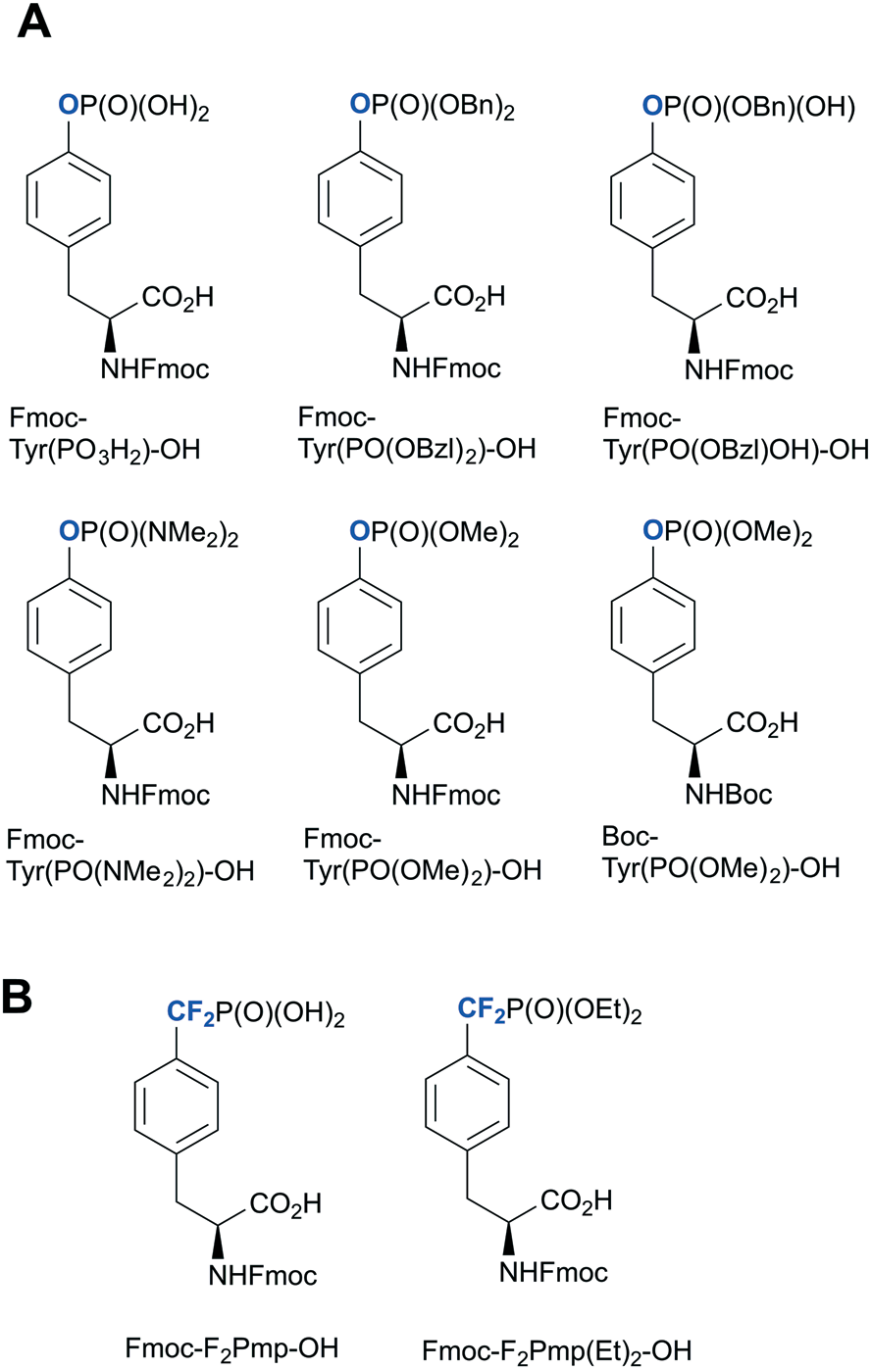
Commercially available phosphotyrosine (A) and phosphonodifluoromethyl phenylalanine (F_2_pmp) (B)-based building blocks.

Over the past decade, several phosphotyrosine building blocks and phosphotyrosine phosphonate-based mimetics have become commercially available,^45^ and are widely employed in peptide synthesis^20,30,46^ (Fig. 2). This represents a major advance from the last decade, however, these compounds remain relatively expensive and mainly suitable only for solid phase peptides synthesis (SPPS). Currently Fmoc-Tyr(PO_3_H_2_)-OH is rarely used in peptide chemistry because of the synthetic problems caused by unprotected P–OH groups.^45^ These problems could be solved by employing bis-protected di-*O*-benzyl/methyl phosphotyrosine building blocks. However, the undesired removal of the methyl or benzyl groups by piperidine during Fmoc-SPPS has been observed.^47^*O*-Phosphate monodealkylation could be decreased by utilizing the non-nucleophilic base, DBU, in a concentration of 2% in DMF for Fmoc-deprotection instead of piperidine.^47^ Despite the presence of a free P–OH group, Fmoc-Tyr(PO(OBzl)OH)-OH is the most frequently used building block. Slow and incomplete incorporation of Fmoc-Tyr(PO(OBzl)OH)-OH during peptide assembly can be resolved by tuning reaction conditions.^48^ Fmoc-Tyr(PO(NMe_2_)_2_)-OH has recently become commercially available facilitating peptide synthesis and allowing mild conditions for phosphate deprotection.

**Scheme 1:**
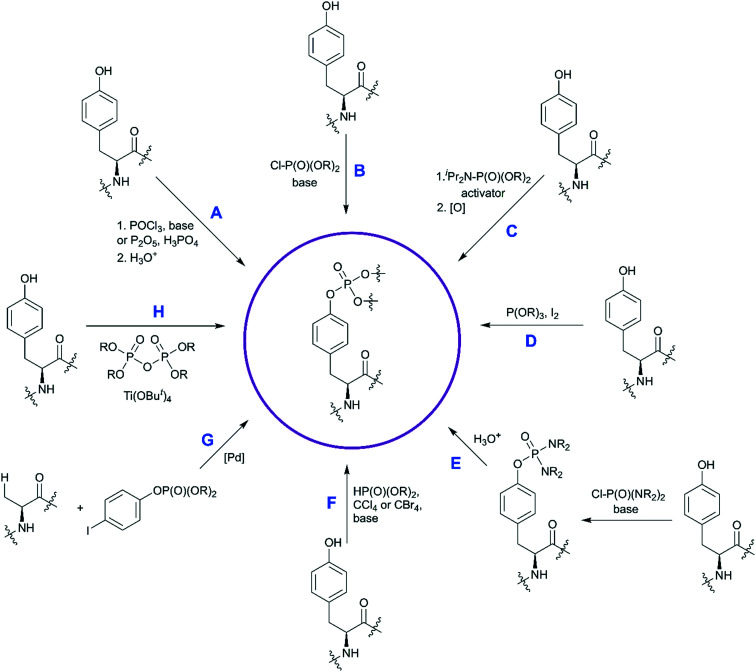
Synthetic strategies for the synthesis of phosphotyrosine. Available methods to synthesize a pY building block are shown. Methods A–C are the most established, and have been discussed in ref. 35. Methods D–H capture more recent developments and are described in detail here.

The custom synthesis of the pY core is based on the phosphorylation of tyrosine and typically achieved using three traditional methods (Scheme 1, paths A–C).^35^ The chemistry of these transformations remained virtually unchanged for decades, yet a few modifications were recently introduced.

**Fig. 3:**
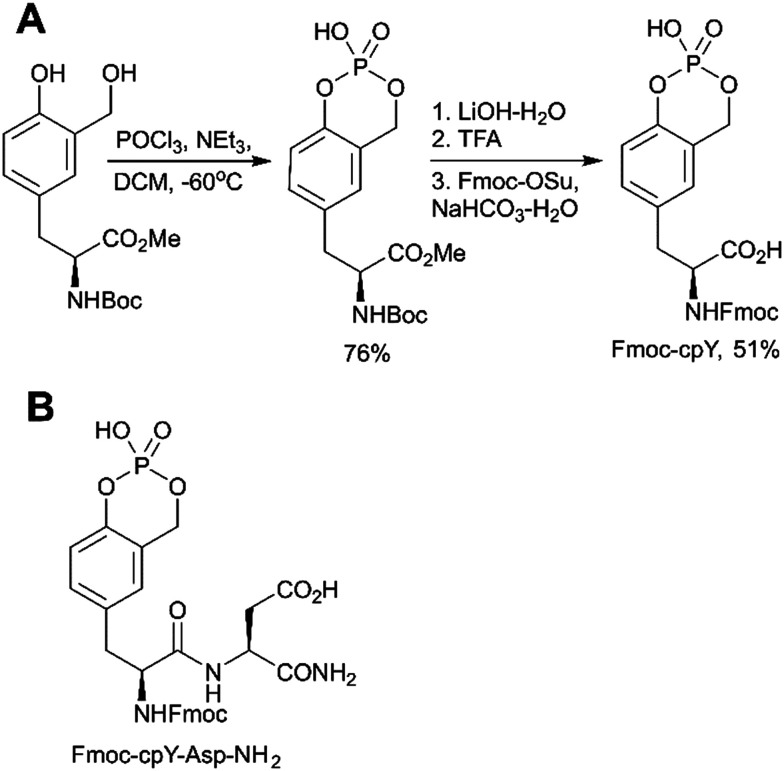
Cyclosaligenyl phosphotyrosine mimetic targeting SAP–SLAM interactions. A) Synthesis of the Fmoc-cpY building block.^52^ B) Chemical structures of the SAP–SLAM inhibitor Fmoc-cpY-Asp-NH_2_.^52^

The first approach is based on introducing the phosphate group using inorganic reagents, POCl_3_ or P_2_O_5_ (Scheme 1, path A). Regardless of harsh conditions, these methods were recently utilized for the synthesis of Fmoc-Tyr(PO_3_H_2_)-OH,^23,49,50^*O*-phospho-3,5-difluorotyrosine^51^ and cyclosaligenyl phosphodiester moiety (Fig. 3A).^52^ Chu *et al.* proposed the cyclosaligenyl phosphodiester (cpY) as a novel phosphotyrosine mimetic and showed that cpY-containing dipeptides inhibited the interaction between SLAM (signaling lymphocytic activation molecule) and SH2-containing SAP (SLAM-associated protein).^52^ SLAM and SAP are involved in immune cell interactions and considered as potential targets for autoimmune disease therapy. Among all obtained dipeptides in this study, Fmoc-cpY-Asp-NH_2_ (Fig. 3B) showed the most potent inhibitory activity with IC_50_ = 17 μM in a cell-free assay system. Interestingly, it was observed that not only pY but also a Fmoc group was required for binding of the synthesized dipeptides, as inhibitor activity was lost after Fmoc group removal.^52^

**Scheme 2:**
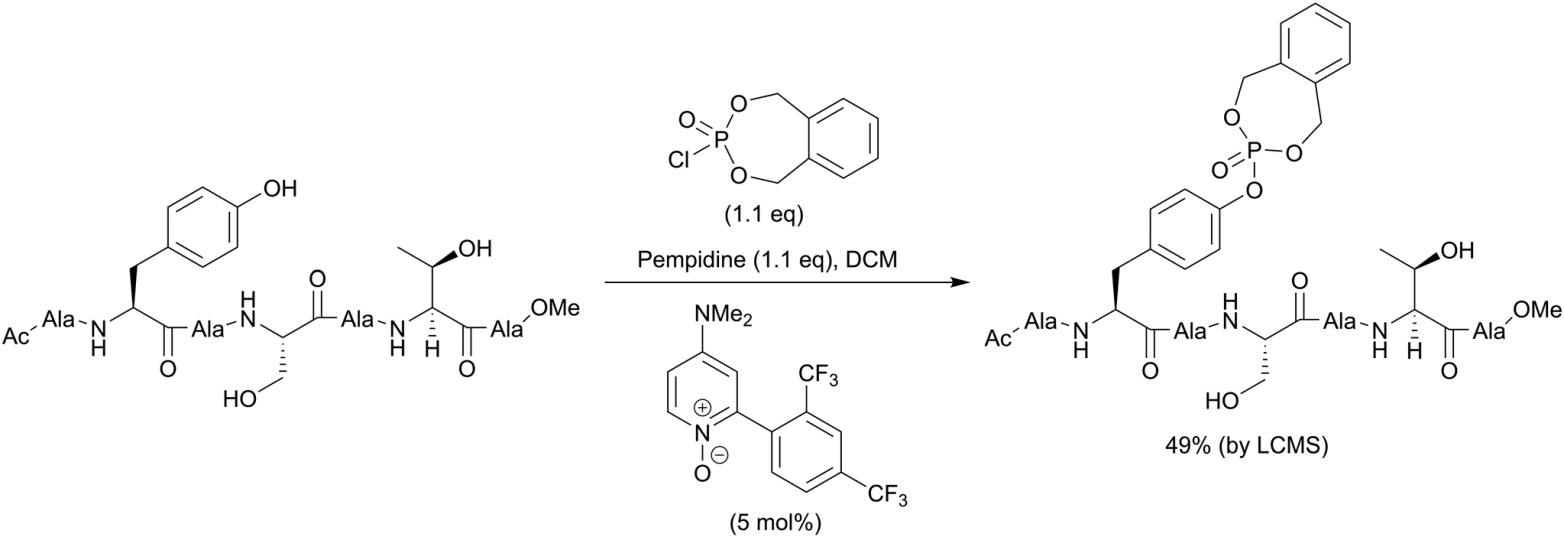
Site-selective phosphorylation of a tyrosine-containing peptide.^55^

The second method is phosphorylation with phosphorohalidates in the presence of base (Scheme 1, path B). Due to the low reactivity of phosphorochloridates, the addition of the catalyst (*e.g.* DMAP or Lewis acids) and/or prolonged reaction time are often required to facilitate the reaction. The latest examples of this method were realised in the synthesis of *O*-diethylphospho l-tyrosine *N*-carboxyanhydride (a monomer for the production poly(l-phosphotyrosine) polymer)^53^ and in the synthesis of the precursor for visible light-induced borylation.^54^ As far as selectivity is concerned, recently Murray and co-workers reported the site-selective phosphorylation of Tyr in the presence of Ser and Thr residues in peptides using *o*-xylenyl phosphoryl chloride,^55^ which makes this method valuable for a post-synthetic global phosphorylation approach. The selectivity was achieved by employing 2-aryl-4-DMAP-*N*-oxide as a catalyst and pempidine (1,2,2,6,6-pentamethylpiperidine) as a base (Scheme 2). Xylenyl phosphate can be subsequently deprotected by hydrogenolysis (H_2_, Pd/C).

A recent improvement in the phosphorohalidates approach allows to generate the dialkyl phosphoriodidate *in situ* from phosphite and iodine (Scheme 1, path D). This relatively inexpensive and convenient method was utilized for the introduction of ethyl and allyl protected phosphates into the tyrosine core.^56–58^ The suggested mechanism involves the Arbuzov reaction of alkyl phosphite with iodine followed by direct phosphorylation of phenol in the presence of base.^58^

**Fig. 4:**
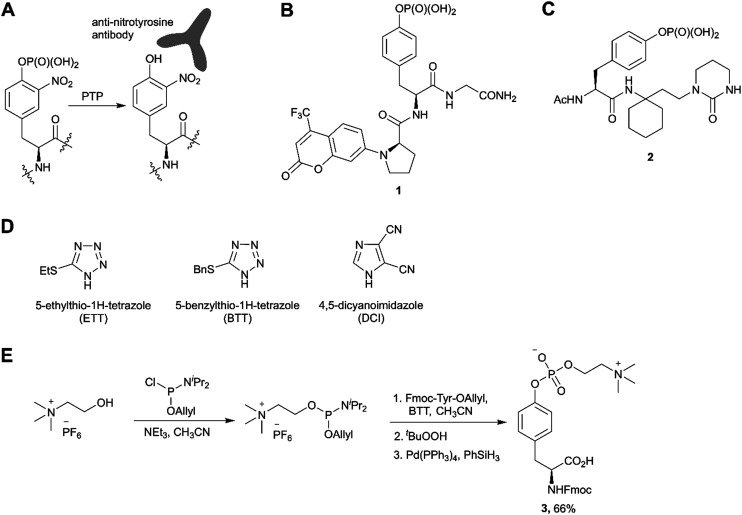
Recent applications and advances in the phosphoramidite approach. A) Strategy of the 3-nitrophosphotyrosine based assay for phosphatase activity measurement.^59^ B) Fluorescence probe 1 for monitoring the phosphatase activity.^15^ C) Grb2-SH2 domain inhibitor 2.^60^ D) Structure of activators alternative to 1*H*-tetrazole. E) Synthesis of phosphocholinated tyrosine building block 3 for Fmoc SPPS.^65^

The third most commonly used approach for tyrosine phosphorylation is based on phosphitylation using phosphoramidites in the presence of an activator, followed by oxidation of P(iii) to P(v) (Scheme 1, path C). A well-established protocol includes tetrazole as activator and oxidation with *t*-butyl hydroperoxide. Recently, this methodology was successfully applied to the development of probes for monitoring PTPs activity. van Ameijde *et al.* designed an assay to monitor dephosphorylation of 3-nitrophosphotyrosine-containing peptides.^59^ In that assay, phosphatase activity is measured *via* formation of 3-nitrotyrosine residue that can be detected by the specific, sequence-independent anti-nitrotyrosine antibody HM11 (Fig. 4A). Another PTP activity probe, 1, was suggested by Choi *et al.* and based on a fluorescence change upon dephosphorylation (Fig. 4B).^15^ Changes on the emission intensity were explained by tyrosine-induced quenching of coumarin fluorescence, albeit *via* a still elusive mechanism. Tyrosine phosphorylation by dibenzyl diisopropylphosphoramidite was used in the development of non-peptide Grb2-SH2 ligands.^60^ Growth factor receptor-bound protein 2 (Grb2) is an adaptor protein between cell membrane receptors and cytoplasmic kinases and has been associated with breast and bladder cancers. Grb2 inhibition blocks the human epidermal growth factor receptor 2 (HER2) activation and therefore is pursued as a drug discovery strategy.^61^ Using molecular modelling and NMR-guided approach, Grb2-SH2 binder 2 (Fig. 4C) was developed and shown to block Grb2 substrate binding with IC_50_ = 56 μM, and to inhibit the proliferation of the HER2-positive MCF7 breast cancer cell line (IC_50_ = 100 μM), with no effect on fibroblasts or HER2-negative breast cancer cell lines.^60^ A similar reaction between tyrosine and bis(*o*-nitrobenzyl)-diisopropylphosphoramidite was utilised for the synthesis of bis(*o*-nitrobenzyl)-phosphotyrosine, which was used for the modification of a suppressor tRNA_CUA_ for the *in vitro* synthesis of proteins with predetermined positions of phosphorylated tyrosine residues.^62^

In phosphoramidite chemistry, 1*H*-tetrazole is most commonly used as activator. Unfortunately, tetrazole exhibits explosive properties and is no longer commercially available in a solid form or an effective concentration due to limited solubility. Several alternative activators (Fig. 4D), such as DCI, ETT, BTT have been developed, which increase the rate and efficiency of phosphoramidite coupling, and are employed in the synthesis of nucleotides. Recently this experience was translated into the synthesis of phosphotyrosine derivatives.^63,64^ Of note, the use of DCI allowed to produce Fmoc-Tyr(PO(OBzl)_2_)-OH in multi-gram scale with an excellent yield.^14^

An interesting example of the use of the phosphoramidites technique was demonstrated by Albers and Hedberg in the preparation of building block 3 (Fig. 4E) for Fmoc SPPS of peptides containing phosphocholinated residues.^65^ In this work, the authors employed 5-(benzylthio)-1*H*-tetrazole (BTT) as the activator. Phosphocholination is a recently discovered post-translational modification important for *Legionella pneumophila* pathogenesis.^66^ Synthetic phosphocholinated peptides are promising tools for studying of phosphocholination/dephosphocholination pathways and raising epitope-specific antibodies for phosphocholinated proteins.

**Scheme 3:**
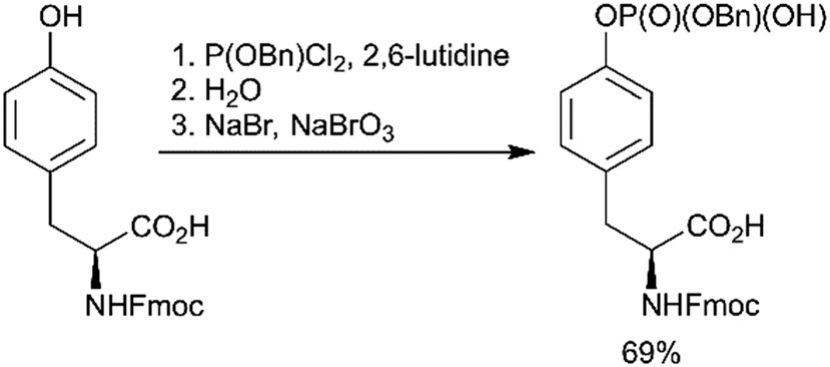
Novel one-pot synthesis of Fmoc-Tyr(PO(OBzl)OH)-OH.^67^

Using a strategy that is conceptually related to the phosphitylation approach described above (Scheme 1, path C), a new efficient one-pot synthesis of protected phosphoamino acids was developed.^67^ One example of products achieved with this route is Fmoc-Tyr(PO(OBzl)OH)-OH (Scheme 3). This compound was previously synthesised in a multistep procedure that included the protection of the carboxylic group,^68^ a step not required by the new method.

**Fig. 5:**
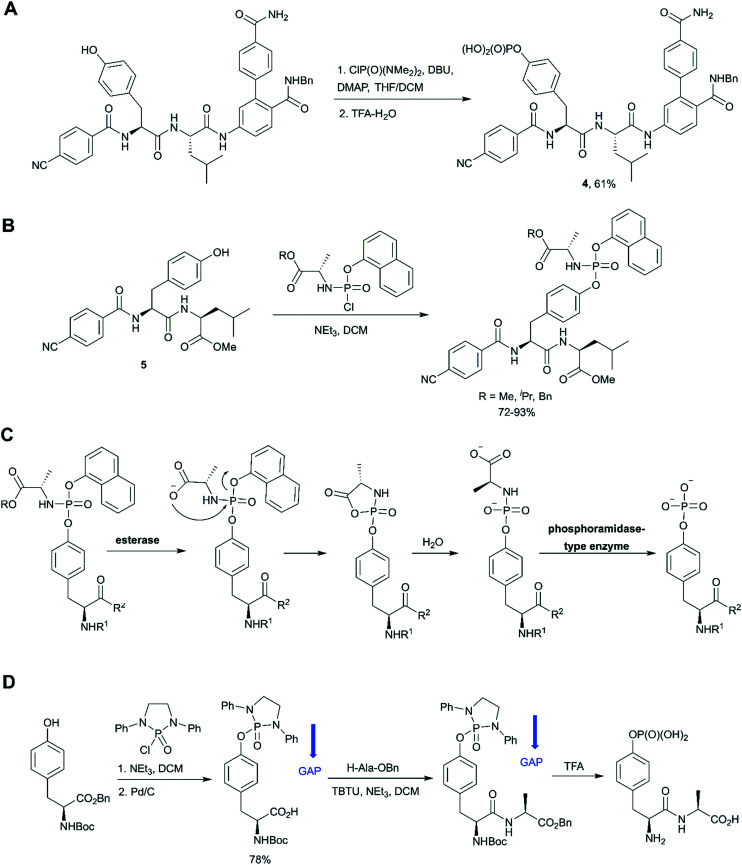
Aminophosphoryl chloride approach in phosphotyrosine synthesis. A) Use of ClP(O)(NMe_2_)_2_ in the synthesis of STAT3 inhibitor 4.^71^ B) Synthesis of aryloxy phosphoramidate prodrugs of ISS-610 (a STAT3 inhibitor).^72^ C) Mechanism of *in vivo* metabolism of the aryloxy phosphoramidate prodrug. D) Group assisted purification (GAP) synthesis of a phosphotyrosine-containing dipeptide.^74^

In the last decade a new trend in the use of aminophosphoryl chlorides in the synthesis of phosphotyrosine has emerged (Scheme 1, path E). Originally reported by Chao and co-workers in 1995,^69^ a method for tyrosine phosphorylation with commercially available bis(dimethylamino)phosphoryl chloride did not receive much attention until recent years. The method allows to introduce *N*,*N*-dialkyldiamide-type phosphate protecting group, which can be easily converted into the corresponding phosphate under mild conditions (method A: 2M HCl/dioxane = 1/1 in 6 h;^69^ method B: 0.04 M HCl (pH 2) in 36 h).^70^ This approach was successfully employed in the synthesis of a spy molecule for ^19^F NMR displacement assays to study pY-peptides binding to SOCS2,^14^ as well as to obtain inhibitors of STAT3 protein (Fig. 5A).^71^ The signal transducer and activator of transcription 3 (STAT3) is an attractive cancer therapeutic target due to its hyperactivation in many human cancers.^8^ STAT3 upregulation stimulates cell proliferation and apoptosis evasion in cancer cells. STAT3 plays a key role in cell signalling from cell membrane receptors into the nucleus. Upon receptor activation by cytokine or growth factor, STAT3 is recruited from the cytoplasm *via* interactions of the STAT3 SH2 domain with specific pY sites of the receptor. The docked STAT3 is then phosphorylated causing its dimerization and nuclear translocation. Since the dimerization of STAT3 is critical for its activation, targeting the STAT3-SH2 domain by small molecules, which block its interaction with receptors and homodimerization, is a promising approach for therapeutic intervention. To design STAT3 inhibitors, Shahani *et al.* fused the structure of previously described inhibitor ISS-610 with a peptide motif derived from the gp130 receptor known to bind the STAT3 SH2 domain.^71^ The synthesised compound 4 (Fig. 5A) showed high affinity to STAT3 (*K*_D_ = 900 nM, by SPR), disrupted the STAT3-gp130 phosphopeptide interaction (IC_50_ = 5 μM) and inhibited STAT3 homodimerization in cells.^71^ However, cell proliferation assay showed only 50% suppression in cell viability of human prostate, pancreatic and breast cancers probably due to poor cell permeability and stability of the inhibitors.

The aminophosphoryl chloride approach was used by Miccoli *et al.* for the preparation of aryloxy triester phosphoramidate prodrugs of ISS-610 obtained by treatment of appropriate tyrosine derivative 5 with (chloro(naphthalen-1-yloxy)phosphoryl)-l-alaninates using triethylamine as a base (Fig. 5B).^72^ This was the first application of the aryloxy phosphoramidate prodrug technology for pY-containing molecules. This technology (so-called ProTide) is widely used in the synthesis of nucleotide prodrugs, including several FDA-approved drugs and clinical candidates, such as sofosbuvir and remdesivir.^73^ In the proposed aryloxy phosphoramidate prodrug approach, the negative charge of the phosphotyrosine is masked by a naphthyl group and an amino acid ester to increase cell membrane penetration. Inside cells the masking groups are cleaved by two consecutive enzymatic activities: first an esterase and second a phosphoramidase activity (Fig. 5C). The choice of the naphthyl rather than phenyl as the aryl motif was critical because naphthyl is a better leaving group than phenol, which prevents the release of the phenol of the phosphotyrosine during metabolism.^72^

Wu and co-workers gave a new impetus to the aminophosphoryl chloride approach by introducing a group assisted purification (GAP) strategy for the synthesis of pY and pY-containing peptides.^74^ GAP chemistry involves the introduction into the molecule of special auxiliaries which generate a compound of adequate solubility. The compound generated is soluble in several solvents (usually DCM, THF, MeOH), but has poor solubility in petroleum solvents and their cosolvents. That is why the desired product can be isolated by a simple filtration and washing. In the suggested approach tyrosine was phosphorylated by 2-chloro-1,3-diphenyl-[1,3,2]diazaphospholidine 2-oxide in the presence of triethylamine to introduce the GAP auxiliary.^74^ The proposed GAP concept allows solution phase peptide synthesis without using chromatography or recrystallization (Fig. 5D). Furthermore, *N*,*N*- diphenylethylenediamine auxiliary can be recovered for re-use. It was suggested that group assisted purification method can complement SPPS, especially for large-scale peptide synthesis. For example, this approach was successfully applied for the synthesis of biphalin peptides.^74^

**Fig. 6:**
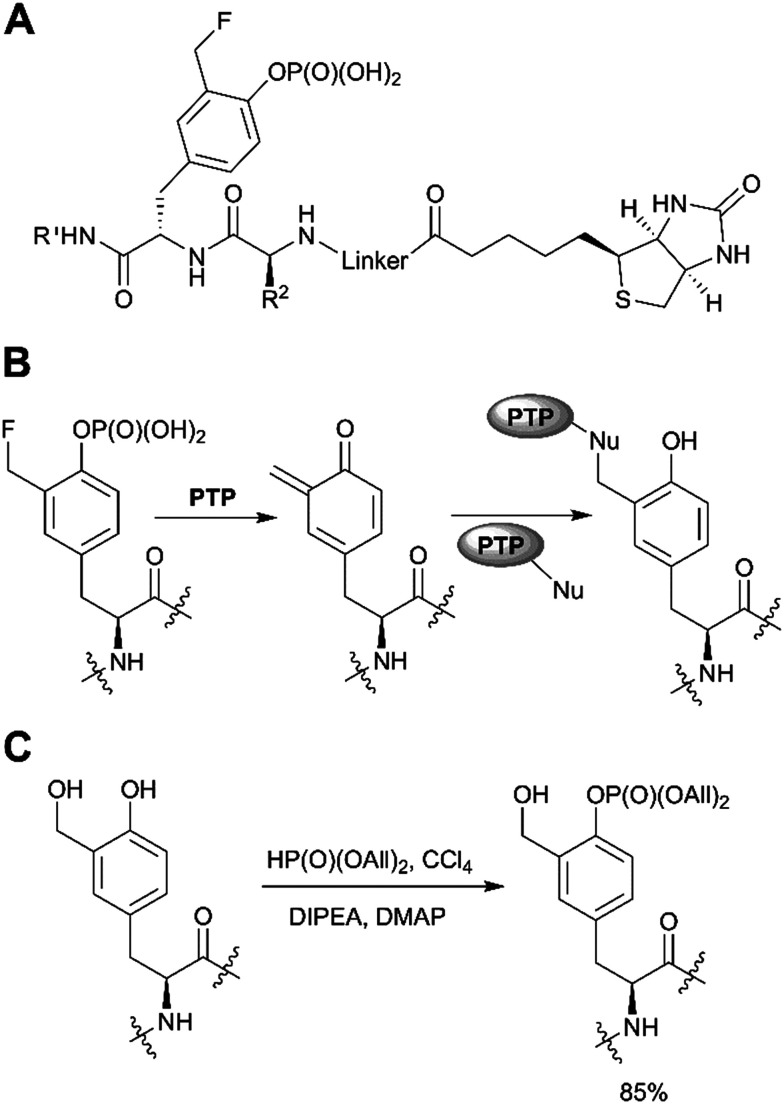
Recent applications of the dialkyl phosphite approach. A) PTP activity-based probes.^16^ B) Proposed mechanism for PTP trapping. C) Selective phosphorylation of the phenolic hydroxyl group by diallylphosphite.^16^

A distinct approach to the synthesis of pY derivatives is based on dialkyl phosphites and can be considered as an extension of the Todd reaction (Scheme 1, path F). It is postulated that tyrosine is phosphorylated by dialkyl halogen phosphate generated *in situ* from dialkyl phosphite and tetrabromo- or tetrachloromethane in the presence of base. This approach was proposed by Szardenings *et al.* for the preparation of phosphotyrosine mimetics using CBr_4_/HP(O)(OEt)_2_/NEt_3_.^75^ Recently this method was used in the synthesis of activity-based probes for protein tyrosine phosphatase, with minor alterations.^16^ The probes consist of a 3-fluoromethylphosphotyrosine warhead connected through a PEG linker to biotin, which served as reporter unit (Fig. 6A). According to the proposed mechanism, after phosphatase hydrolysis the probe undergoes elimination of the fluorine yielding an intermediate which reacts with nucleophilic residues of PTP (Fig. 6B). After formation of a covalent bond the enzyme can be detected using anti-biotin antibody. 3-Fluoromethylphosphotyrosine warhead was incorporated into peptides that were used to study the substrate specificity of PTPs.^16^ Of note, the use of dialkyl phosphites allows a selective phosphorylation of the phenolic hydroxyl group (Fig. 6C).^16^

**Scheme 4:**
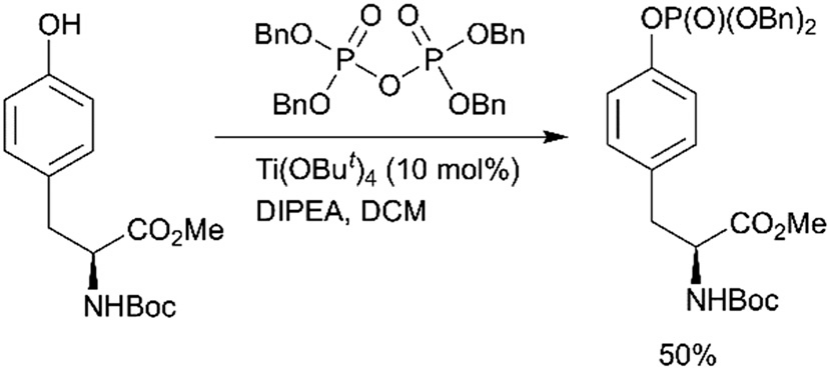
Lewis acid catalysed tyrosine phosphorylation with pyrophosphates.^76^

Fenton *et al.* reported the Lewis acid catalyzed phosphorylation with pyrophosphates (Scheme 1, path H).^76^ Titanium(iv) *tert*-butoxide was found to be the most effective catalyst for the reaction of Boc-Tyr-OMe with tetrabenzylpyrophosphate (Scheme 4). Other pyrophosphates with methyl, ethyl, allyl, and *o*-nitrobenzyl protecting groups are synthetically available and might serve as phosphorylating agents.

**Scheme 5:**
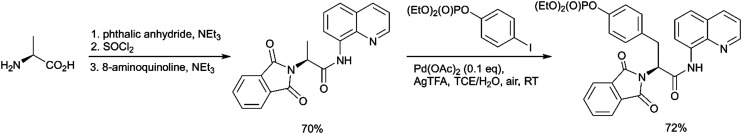
Palladium-catalyzed mono-arylation of the β-methyl group of alanine.^77^

In contrast to the above-described methods for pY synthesis based on tyrosine phosphorylation, Wang *et al.* demonstrated a new strategy – palladium-catalyzed *N*-quinolylcarboxamide directed arylation of the unactivated β-C(sp^3^)–H bonds of alanine (Scheme 1, path G).^77^ Phthaloyl alanine reacts with (4-iodophenyl)diethylphosphate under the developed conditions providing the desired pY-containing compound in good yield (Scheme 5). The 8-aminoquinoline auxiliary group is essential for the Pd-catalyzed C(sp3)–H bond activation and the use of silver trifluoroacetate in 1,1,2,2-tetrachloroethane (TCE) at room temperature allows to perform mono-selective arylation of alanine. Interestingly, the reaction is tolerant to both water and air. The starting phthaloyl alanine bearing 8-aminoquinoline group was obtained in three steps from l-alanine. The 8-aminoquinoline auxiliary can be cleaved under mild conditions by treatment with LiOH–H_2_O_2._^77^

Although this approach has been demonstrated only for generating of a pY core group, it can be readily expanded to the synthesis of different pY mimetics using the corresponding aryl iodides.

## Advances in the synthesis of phosphonate-based phosphotyrosine mimetics

**Scheme 6:**
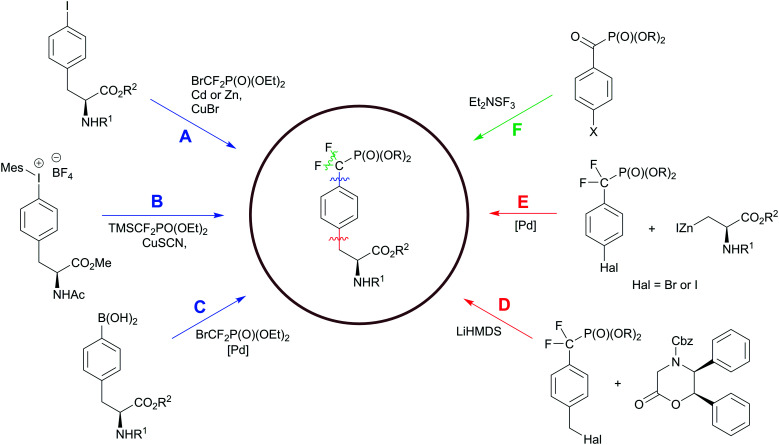
Synthetic strategies to the phosphonodifluoromethyl phenylalanine. Paths D–F have been discussed in the ref. 36. Colour scheme represents the bond formation: C–F bond, green; Ar–CH_2_, red; CF_2_–Ar, blue.

Hydrolysis of the pY phosphate group by PTPs is a key step in cell signalling, but makes phosphotyrosine a liable functional group in the design of SH2, PTB or PTP inhibitors. Non-hydrolysable pY mimetics are thus well-established and their synthesis has been reviewed.^36^ Among all developed phosphonate-based phosphotyrosine mimetics, in the last decade phosphonodifluoromethyl phenylalanine (F_2_pmp) received much attention due to its structural features and physicochemical properties, such as a similar p*K*_a_ compared to pY, and the presence of the methylene fluorine atoms, which may mimic the hydrogen bond interactions of phenolic oxygen with active site residues. Therefore, the interest in F_2_pmp stimulates the ongoing development of novel synthetic approaches to F_2_pmp derivatives (Scheme 6).

**Fig. 7:**
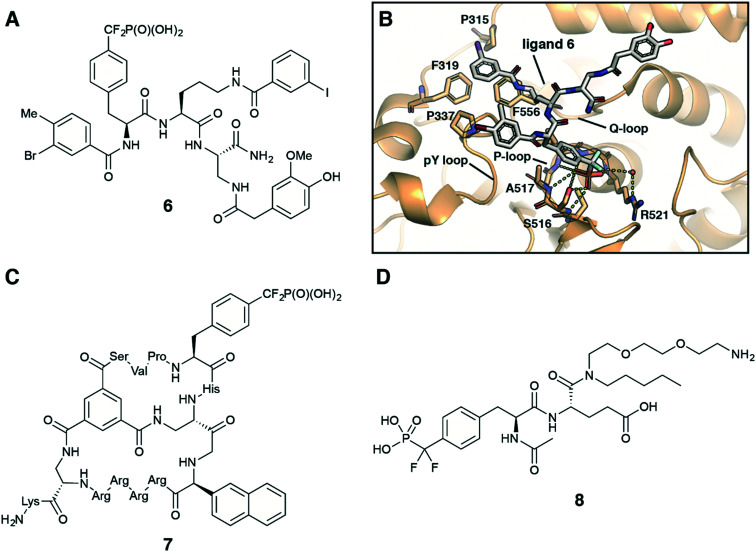
Recent application of F_2_Pmp in drug design. A) Chemical structure of PTP-MEG2 selective inhibitor 6.^32^ B) Crystal structure of PTP-MEG2 with inhibitor 6 (PDB code: 4GE6).^32^ C) Chemical structure of cell-permeable bicyclic peptide PTP1B inhibitor 7.^31^ D) Chemical structure of ligand 8 for the inhibitor affinity purification of SH2 proteins.^33^

Fmoc-F_2_Pmp-OH is widely utilized in the synthesis of F_2_Pmp-containing peptides and small molecules for use as inhibitors against PTPs.^12,30–32^ Considering pY as a main substrate for PTPs, it is a common strategy in the ligand design to employ a nonhydrolyzable pY mimetic, such as F_2_Pmp, which occupies the active site with the same binding mode as pY. Introducing molecular diversity at C- and N-terminal sides of F_2_pmp achieves high binding affinity and selectivity by targeting specific flanking residues in the pocket. Zhang *et al.* followed this logic in the development of a potent and selective inhibitor for megakaryocyte protein tyrosine phosphatase 2 (PTP-MEG2) (Fig. 7A).^32^ Ligand 6 was synthesised using standard solid-phase Fmoc chemistry on a Rink amide resin. Compound 6 inhibited PTP-MEG2 with IC_50_ = 75 nM, showed more than 18-fold selectivity for PTP-MEG2 over PTP1B and T-cell protein tyrosine phosphatase (TC-PTP), and exhibited no inhibition of any other tested mammalian PTPs. The co-crystal structure of PTP-MEG2 with inhibitor 6 revealed that F_2_Pmp fragment, as anticipated, bound to the active site pocket of the enzyme making contacts with residues in the pY recognition loop, P-loop and Q-loop (Fig. 7B). At the same time 3-iodobenzoic amide moiety binds to a hydrophobic groove created by Pro315, Phe319, Pro337, and Phe556 residues, which are unique to PTP-MEG2 (Fig. 7B). These less conserved interactions were thought to be responsible for the inhibitor selectivity and potency. Furthermore, 3-bromo-4-methylbenzoic amide, homovanillic amide and diaminopropionic acid linker make additional contacts with the enzyme, contributing to the high binding affinity. Targeting PTP-MEG2 attracts considerable attention as new approach for the treatment of type 2 diabetes due to its negative regulation of insulin signalling. Indeed, the developed inhibitor improved insulin sensitivity and glucose homeostasis in diet-induced obese mice.^32^ Interestingly, compound 6 showed efficient cellular and *in vivo* activity despite the presence of a charged phosphonate group.

Recently, Pei with co-workers employed the F_2_pmp scaffold in the design of cell-permeable bicyclic peptides targeting PTPs.^30,31^ To obtain potent, selective and active *in vivo* inhibitors of PTPs the authors fused two peptide rings. One ring is a F_2_Pmp-containing cyclic peptide, which is responsible for target binding. The other ring is a cell-penetrating peptide contributing to cellular entry. For example, molecule 7 (Fig. 7C) showed 16-fold selectivity for PTP-1B (with IC_50_ = 30 nM) over TC-PTP and no inhibition against a large panel of tested PTPs.^31^ Moreover, the authors demonstrated that compound 7 efficiently entered human cells A549 and inhibited PTP1B activity *in vivo*.

**Fig. 8:**
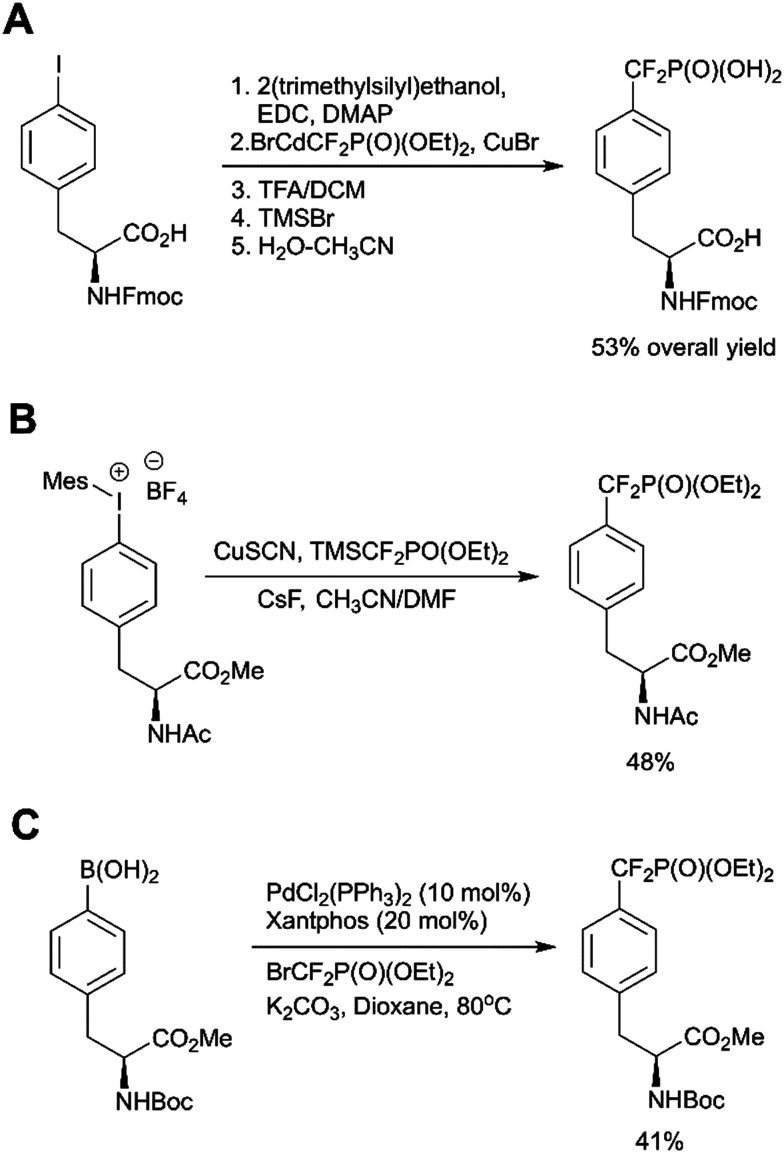
Recent advances in the F_2_Pmp synthesis. A) Optimised synthesis of Fmoc-F_2_Pmp-OH.^78^ B) Copper-mediated synthesis of AcNH-F_2_Pmp-OMe from an iodonium salt.^79^ C) Palladium-catalyzed difluoroalkylation of Boc-4-borono-l-phenylalanine.^80^

In recent years Fmoc-F_2_Pmp-OH has become commercially available (Fig. 2), but it is still relatively costly. To address this issue, Meyer and Köhn optimised Qabar's approach (Scheme 6, path A) for a fast and efficient synthesis of Fmoc-F_2_Pmp-OH.^78^ This method is based on the copper(i) catalysed reaction between phosphonodifluoromethyl-cadmium bromide and Fmoc-Tyr(I)-OTMSE (Fig. 8A). CuBr was shown as superior to CuCl in facilitating this transformation. The final product can be obtained in gram scale.

A similar copper-mediated cross coupling of phosphonodifluoromethyl-zinc bromide was employed to make F_2_Pmp-containing building block 8, which was used for the production of an affinity resin to enrich SH2 proteins from cell lysates.^33^ To perform the inhibitor affinity purification, ligand 8 (Fig. 7D) was immobilized on NHS-activated Sepharose. Following pull-down from cell lysates, 22 out of 55 SH2 proteins were enriched.

Ivanova *et al.* reported a new copper catalysed formation of aryl difluoromethylphosphonates from iodonium salts and TMSCF_2_PO(OEt)_2_ in the presence of CsF.^79^ This methodology was successfully applied to a synthesis of AcNH-F_2_Pmp-OMe (Fig. 8B). Carrying out the reaction in a glovebox and multistep preparation of the starting material make this approach less attractive in comparison to traditional methods.

Common methods for the synthesis of F_2_Pmp derivatives involve the fluorination of corresponding ketophosphonates with aminosulfur trifluorides or the introduction difluoromethylated phosphonate group *via* copper-mediated cross-couplings (Scheme 6, paths A, E and F).^36^ However, these approaches are suboptimal because they are either incompatible with numerous functional groups or use a large excess of a toxic cadmium precursor BrCdCF_2_P(O)(OEt)_2_. To overcome these drawbacks, Feng *et al.* proposed the palladium-catalyzed difluoroalkylation of the commercially available Boc-4-borono-l-phenylalanine methyl ester with bromodifluoromethylphosphonate by using a catalytic system consisting of Pd(PPh_3_)_4_, xantphos and K_2_CO_3_ in dioxane (Fig. 8C).^80^ The choice of the ligand, base and solvent was found to be crucial to achieve reaction efficiency. 4-Boronophenylalanine analogues are commercially available or synthetically accessible. They can be readily synthesised from 4-iodophenylalanine precursors or tyrosine derivatives *via* triflates. In light of this, Feng's approach (Scheme 6, path C) may allow not only to produce a broad range of derivatives, but also to generate molecular matched pairs useful to build structure–activity relationships (SAR) in inhibitor development campaigns.

**Fig. 9:**
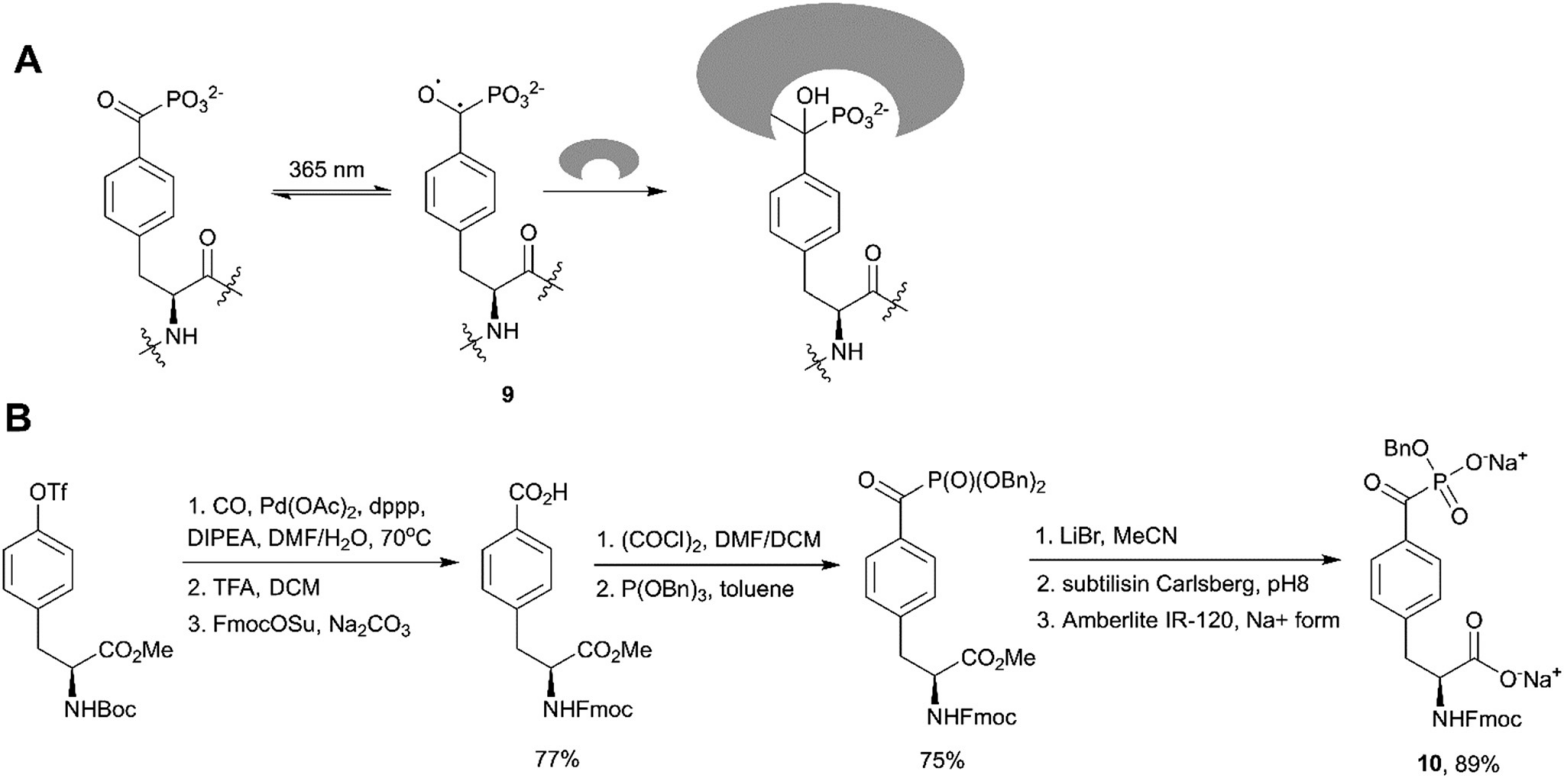
Benzoylphosphonates as phosphotyrosine mimetics. A) Proposed mechanism for photoactivation of 4-phosphonocarbonyl phenylalanine and crosslinking with protein binding sites.^81^ B) Synthesis of 4-phosphonocarbonyl phenylalanine-containing building block 10 for Fmoc SPPS.^81^

Fluorination of corresponding benzoylphosphonates was one of the first approaches toward F2Pmp derivatives (Scheme 6, path F).^36^ Recently Rademann and co-workers demonstrated that α-ketophosphonates, and particularly 4-phosphonocarbonyl phenylalanine (pcF), can also be used as photoactive enzymatically stable phosphotyrosine mimetics targeting, deactivating and labelling phosphotyrosine binding proteins.^81–83^ For example, pcF was incorporated into the sequence of a STAT5 SH2 domain-directed peptide, labelled with 5,6-carboxyfluorescein (CF) for binding studies and protein detection.^81^ The resulting peptide 5-CFGpcFLSLPPW-NH_2_ exhibited 20-fold reduced binding affinity in comparison to a native phosphotyrosine-containing peptide. Irradiation of this peptide at 365 nm resulted in a formation of a triplet biradical intermediate 9 of the benzoylphosphonate moiety leading to a covalent modification of target proteins (Fig. 9A).^81^ Recognition by a phosphotyrosine binding pocket allowed photo-crosslinking with high specificity. This approach was utilised to pull-down STAT5 from cell lysates using a similar pcF-containing, but dual-labelled (carboxyfluorescein and biotin) STAT5-SH2-binding peptide, and was applied to confirm selectivity of the studied inhibitors.^82^

To integrate pcF into peptides, Horatscheck *et al.* developed building block 10 for Fmoc SPPS (Fig. 9B).^81^ Compound 10 was obtained *via* Michaelis–Arbuzov acylation of tribenzyl phosphite with the corresponding carboxylic acid chloride. It is noteworthy that the authors produced the monobenzyl phosphonate foreseeing a P–C bond cleavage of the dialkyl acylphosphonate moiety by nucleophiles such as piperidine during peptide synthesis. In addition, the carboxylic acid was converted into its sodium salt to increase the stability of the final product.

Wagner *et al.* reported an application of pcF-containing peptides as potent, light-switchable inhibitors of the protein tyrosine phosphatase PTP1B.^83^ The phosphopeptide mimetics were found to be moderate inhibitors with Ki-values in the micromolar range. However, irradiation of the pcF-containing peptides with 365 nm light in the presence of PTP1B enhanced inhibitory activity up to 120-fold. Deactivation of PTP1B was demonstrated to proceed *via* an oxidative radical mechanism and could be reverted by addition of dithiothreitol as a reducing agent. Interestingly, the observed effect of PTP1B deactivation by irradiated benzoylphosphonates without covalent labelling of the target protein is distinct from the reported photo-crosslinking of STAT5-SH2 domain.^81^

## Conclusions and future perspectives

In this review we have summarized recent progress in the synthesis of phosphotyrosine-containing compounds. Since the discovery of tyrosine phosphorylation in 1979, pY-containing compounds have attracted considerable attention due to their biological relevance and importance for targeting protein phosphorylation pathways. The last decade has seen the emergence of commercially available pY-containing building blocks for SPPS, which aided peptide synthesis and almost completely superseded post-synthetic global phosphorylation approach.

For many years the synthesis of pY-containing small molecules has been standing on three synthetic pillars (Scheme 1, path A–C).^35^ Recently, many efforts have been made to improve these methods and develop new ones. Early approaches of tyrosine phosphorylation using inorganic reagents are fading away, since they require harsh conditions and are incompatible with many functional groups. The phosphoramidite route still dominates the field and is likely to remain mainstream, taking into the account the introduction of alternative tetrazole activators and numerous commercially available phosphoramidites. However, the phosphoramidite approach does not allow site selective phosphorylation of a tyrosine hydroxyl group in the presence of other hydroxyl groups. To this end, newly developed strategies provide solutions to this problem (Scheme 2 and Fig. 6C).^16,55^ The emerging aminophosphoryl chloride approach offers not only a promising GAP synthesis,^74^ which can complement the traditional SPPS, but also allows the preparation of aryloxy triester phosphoramidate prodrugs,^72^ thereby expanding the phosphotyrosine prodrug toolbox.

An important aspect to consider during the multi-step synthesis is at what step to choose to introduce the phosphate group (or its analogues). The phosphorus containing functional group can serve as a protecting group. Also, the presence of phosphorus atom helps to monitor the course of a reaction by ^31^P NMR spectroscopy, and the reaction mixture can be analysed without any purification or work-up. On the other hand, the polar dialkylphosphate group can complicate the purification of intermediates, and phosphorylation can interfere with other functional groups in the synthesis.

pY derivatives are useful chemical tools to study protein phosphorylation and dephosphorylation and are promising starting points for inhibitor design. However, their use in drug development are limited owing to enzymatic lability and poor cell membrane permeability. To overcome these limitations, more physiologically stable and less polar pY mimetics have been developed, including the promising F_2_pmp group. Although several approaches towards F_2_pmp have been proposed, limitations remain in their synthesis and general applicability. The introduction of a phosphonodifluoromethyl fragment is a challenging process and has a narrow substrate scope. Therefore, development of methods with mild reaction conditions is much needed and is anticipated to be an area of fertile progress in future.

The poor permeability, stability and bioavailability of pY and F_2_Pmp containing molecules remain major challenges to overcome to unlock their application as inhibitors for biological targets. These challenges can potentially be relaxed and circumvented by new therapeutic modalities such as targeted protein degradation, for example using bifunctional small-molecule PROTACs (proteolysis targeting chimeras).^84–86^ PROTACs hijack the catalytic activity of E3 ubiquitin ligases to induce ubiquitylation and subsequent proteasomal degradation of a protein target. One of the main advantages of using PROTAC degraders over conventional occupancy-based inhibitors is that PROTACs can work sub-stoichiometrically at lower concentrations, due to their catalytic mode of action *via* a trimeric complex.^87,88^ Because PROTACs do not require to fully occupy the target binding site, there are less strict requirements on their cellular permeability or binary binding affinity to the target protein and E3 ligase.^87,92^ In addition, PROTAC trimeric complexes can offer a boost in target degradation potency and selectivity over and above what might be expected from the binding group alone.^88,89^ Therefore, unoptimized pY properties, albeit unacceptable in the context of occupancy-based inhibitors, are predicted to be more tolerated in the context of PROTAC degraders. Indeed, a potent STAT3 PROTAC degrader, SD-36, based on a pY-derivative moiety was recently reported.^90,91^ Despite having unmasked CF_2_P(O)(OH)_2_ group, SD-36 showed efficient cellular and *in vivo* activity inducing potent and selective degradation of STAT3, which resulted in pronounced and long-lasting tumour regression in xenograft mouse models.^90^ We anticipate that the PROTAC approach will give a new momentum to the use of pY and their analogues, and attractive new future directions in the field.

## Conflicts of interest

The Ciulli laboratory receives or has received sponsored research support from Amphista Therapeutics, Boehringer Ingelheim, Eisai, Nurix Therapeutics, and Ono Pharmaceutical. A. C. is a scientific founder, shareholder, director and consultant of Amphista Therapeutics, a company that is developing targeted protein degradation therapeutic platforms. N. M. reports no competing interests.

## Supplementary Material
